# *Porphyromonas gingivalis* Vesicles Control Osteoclast–Macrophage Lineage Fate

**DOI:** 10.3390/ijms27020831

**Published:** 2026-01-14

**Authors:** Elizabeth Leon, Shin Nakamura, Satoru Shindo, Maria Rita Pastore, Tomoki Kumagai, Alireza Heidari, Elaheh Dalir Abdolahinia, Tomoya Ueda, Takumi Memida, Ana Duran-Pinedo, Jorge Frias-Lopez, Xiaozhe Han, Xin Chen, Shengyuan Huang, Guoqin Cao, Sunniva Ruiz, Jan Potempa, Toshihisa Kawai

**Affiliations:** 1 Department of Oral Science and Translational Research, College of Dental Medicine, Nova Southeastern University, Fort Lauderdale, FL 33314, USAedalirab@nova.edu (E.D.A.); kyleliu7569@gmail.com (X.C.);; 2 Department of Periodontics and Endodontics, Division of Dentistry, Okayama University Hospital, Okayama 700-8525, Japan; 3 Department of Oral Biology, College of Dentistry, University of Florida, Gainesville, FL 32610, USA; 4 Department of Oral Immunology and Infectious Diseases, School of Dentistry, University of Louisville, Louisville, KY 40202, USA; 5 Department of Microbiology, Faculty of Biochemistry, Biophysics, and Biotechnology, Jagiellonian University, 30-387 Kraków, Poland

**Keywords:** *Porphyromonas gingivalis*, outer membrane vesicle, periodontitis pathogenesis, macrophage polarization, osteoclastogenesis, OC/MΦ unit

## Abstract

*Porphyromonas gingivalis* (*Pg*), a keystone pathogen of chronic periodontitis, releases outer membrane vesicles (OMVs) that act as nanoscale vehicles to disseminate virulence factors within periodontal tissues and systemically beyond the oral cavity. Although *Pg*-OMVs are increasingly recognized as critical mediators of host–pathogen interactions, their effects on the differentiation and function of monocyte–macrophage/osteoclast lineage cells remain unclear. Here, we examined the impact of *Pg*-OMVs on the differentiation of RAW264.7 monocyte/macrophage-like cells into osteoclasts (OC) and/or macrophages (MΦ) in the presence of receptor activator of nuclear factor-κB ligand (RANKL). OMVs were isolated from *Pg* W83 and applied to RANKL-primed RAW264.7 cells using three distinct stimulation schedules: (1) simultaneous treatment with *Pg*-OMVs and RANKL at Day 0; (2) RANKL priming at Day 0 followed by *Pg*-OMV stimulation at Day 1; and (3) RANKL priming at Day 0 followed by *Pg*-OMV stimulation at Day 3. In all schedules, cells were cultured for 7 days from the initial RANKL exposure. Remarkably, simultaneous exposure to *Pg*-OMVs and RANKL (Schedule 1) markedly suppressed osteoclastogenesis (OC-genesis) while promoting M1 macrophage polarization. In contrast, delayed *Pg*-OMV stimulation of RANKL-primed cells (Schedules 2 and 3) significantly enhanced OC-genesis while reducing M1 polarization. These schedule-dependent effects were consistent with altered expression of osteoclastogenic markers, including *dc-stamp*, *oc-stamp*, *nfatc1*, and *acp5*. Importantly, a monoclonal antibody against OC-STAMP counteracted the *Pg*-OMV-induced upregulation of OC-genesis in Schedules 2 and 3. Furthermore, levels of *Pg*-OMV phagocytosis were inversely correlated with osteoclast formation. Finally, co-stimulation with RANKL and *Pg*-OMVs (Schedule 1) enhanced macrophage migratory capacity, whereas delayed stimulation with *Pg-*OMVs (Schedules 2 and 3) did not. Collectively, these findings indicate that *Pg*-OMVs exert stage-specific effects on the OC/MΦ lineage: stimulation at early stages of RANKL priming suppresses OC-genesis and promotes M1 polarization, whereas stimulation at later stages enhances OC-genesis without inducing M1 differentiation. Thus, *Pg*-OMVs may critically influence the fate of the OC/MΦ unit in periodontal lesions, contributing to disease progression and tissue destruction.

## 1. Introduction

Periodontal disease is an inflammatory disease caused by the complex interplay of oral bacteria and host inflammatory response [1]. The progression of periodontal disease results in destruction of periodontal tissues, including alveolar bone resorption, gingival recession, and tooth loss. *Porphyromonas gingivalis* (*Pg*) is a Gram-negative anaerobic bacterium that is frequently isolated from the periodontal pocket of patients with periodontitis. *Pg* produces various virulent factors, such as gingipains [2], lipopolysaccharides (LPS) [3], and fimbriae [4], all of which exacerbate the damage caused by *Pg* on the periodontal tissue and allow the bacterium to be recognized as the primary pathogen associated with chronic systemic disease risk, including diabetes [5], rheumatoid arthritis [6], and Alzheimer’s disease [7]. In addition, *Pg* can disrupt commensal oral, serum, and gut microbiota, resulting in dysbiosis [8,9]. Therefore, *Pg* is recognized as a keystone pathogen in periodontal disease [10,11].

Outer membrane vesicles (OMVs) are nanosized particles (50–250 nm in diameter) produced by Gram-negative bacteria, such as *Pg* [12]. The production of *Pg*-OMVs begins with the excision of the bacterial cell wall and the accumulation of phospholipids in the outer membrane, which causes its expansion. Linkages between the peptidoglycan and the outer membrane layer are disrupted, and finally, *Pg*-OMVs that are filled with bacterial components are released [13,14]. *Pg*-OMVs are indeed enriched with the bacteria’s virulent factors, including gingipains, fimbriae, and LPS [15,16]. Therefore, owing to their small size, *Pg*-OMVs function as sophisticated vehicles to disseminate their virulent factors to the remote tissues and organs [17]. *Pg*-OMVs can inhibit fibroblast and endothelial cell proliferation, as well as suppress angiogenesis, which results in disrupting wound healing and regeneration of periodontal tissue [18]. Moreover, it has been reported that *Pg*-OMVs induce lung injury by disrupting the barrier function of the lung alveolar epithelium [19] and that *Pg*-OMVs promote the development of diabetes by dysregulating the glucose metabolism in the liver [20], supporting the premise that *Pg*-OMVs are capable of spreading to remote tissues and organs. Therefore, *Pg*-OMVs are believed to participate in the various pathological functions caused by *Pg*.

However, despite bone resorption being the central pathological hallmark of periodontal disease, little is known about how *Pg*-OMVs directly affect osteoclasts, the primary bone-resorbing cells. Osteoclasts play an essential role in physiological bone metabolism [21], arise from monocyte/macrophage-lineage cells derived from hematopoietic stem cells, and differentiate into mature multinucleated osteoclasts through the action of receptor activator of nuclear factor-κB ligand (RANKL) and macrophage colony-stimulating factor (M-CSF) [22]. Disruption of this macrophage-to-osteoclast differentiation process can lead to excessive bone resorption and inflammatory bone destruction, and excessive accumulation of osteoclasts can result in bone lytic diseases, including osteoporosis and periodontal disease [23]. Therefore, understanding how external factors control the entire trajectory of monocyte-to-osteoclast differentiation is crucial for elucidating the pathogenesis of periodontal disease.

Previous studies have demonstrated that *Pg*, its components such as LPS, and inflammatory cytokines including TNF-α, IL-6, and IL-1β can promote osteoclast differentiation through both RANKL-dependent or -independent mechanisms [24,25,26,27,28,29]. *Pg*-OMVs have also been reported to promote osteoclastogenesis (OC-genesis) from RANKL-primed macrophage through toll-like receptor 2 (TLR2)-mediated cell signaling [30]. Such evidence supports the induction and promotion of OC-genesis by *Pg* and its components. In contrast, *Pg*-OMVs strongly induce pro-inflammatory cytokines compared with *Pg* whole cells and could activate the inflammasome, leading to pyroptosis in macrophages [31]. The stimulation of macrophages with *Pg*-OMVs also causes a metabolic shift, which increases the expression of genes associated with glycolysis and decreases the expression of genes related to the TCA cycle [31]. Although *Pg*-OMVs exert substantial effects on both macrophages and osteoclasts, previous studies have not clarified which specific stage of monocyte-derived osteoclast differentiation is targeted by *Pg*-OMVs, how *Pg*-OMVs alter lineage fate determination, or how these changes ultimately influence the bone-resorptive function of osteoclasts.

In osteoclast differentiation systems, a heterogeneous cellular population—comprising mature osteoclasts, osteoclast precursors, and macrophages—collectively forms the osteoclast/macrophage (OC/MΦ) unit [32,33]. Macrophages within this unit can polarize into pro-inflammatory (M1) and anti-inflammatory/tissue-regenerative (M2) phenotypes, depending on the stimuli they encounter [34,35]. Similarly, gingival tissues from patients with periodontitis harbor a diverse mixture of monocytes, macrophages and osteoclasts, most of which originate from monocytes migrating from circulation [36,37]. Given this complex landscape, *Pg*-OMVs may interfere with fate determination from monocytes toward osteoclasts, thereby disrupting the differentiation balance within the OC/MΦ unit. However, no study to date has dissected these effects across individual differentiation stages. Based on this background, the present study aimed to comprehensively examine how *Pg*-OMVs affect each stage of monocyte-derived osteoclast differentiation and how they alter the differentiation balance and functional properties of the OC/MΦ unit, using RAW264.7 cells as a model system (Figure S1). This study presents a new conceptual framework, positioning *Pg*-OMVs not merely as inflammatory stimuli but as lineage-determining factors that reprogram monocyte lineage differentiation itself, thereby advancing our understanding of mechanisms of alveolar bone resorption in periodontal disease.

## 2. Results

### 2.1. Characterization of Pg-OMVs

**Figure 1:**
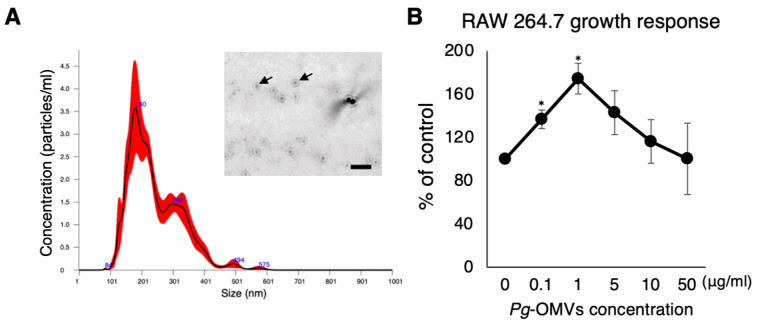
Experimental schedules and characterization of *Pg*-OMVs. (**A**) Representative nanoparticle tracking analysis showing size distribution and morphology of purified *Pg*-OMVs. The peak particle size was ~180 nm with a mean diameter of ~240 nm. The images were obtained with NanoSight nanoparticle tracking software. Scale bar indicates 1 µm. The arrows indicate *Pg*-OMV particles. The red shaded area represents the standard deviation obtained from repeated NanoSight measurements, and the numerical labels indicate the particle diameter. (**B**) Dose–response analysis of *Pg*-OMVs on RAW264.7 proliferation showing no cytotoxicity across 0.1–50 µg/mL, with enhanced proliferation at 1 µg/mL. This concentration was selected for subsequent experiments. Statistical analyses were performed using the Kruskal–Wallis test followed by Steel’s post hoc test for comparisons against the no *Pg*-OMVs group. Each point represents the mean value ± SD (n = 3). Experiments were independently repeated three times with similar results. *: *p* < 0.05, vs. no *Pg*-OMVs.

*Pg*-OMVs were purified from *Pg*’s culture supernatant and characterized using NanoSight nanoparticle tracking software (NTA3.4). The diameter of purified *Pg*-OMVs was 241.9 ± 2.7 nm on average and the peak of the abundant diameter was detected at 182.9 ± 9.5 nm (Figure 1A). *Pg*-OMVs exhibited no cytotoxicity toward RAW264.7 cells at concentrations ranging from 0.1 to 50 µg/mL. Notably, treatment with 1 µg/mL *Pg*-OMVs resulted in the highest level of cell proliferation (Figure 1B). Therefore, a concentration of 1 µg/mL was selected for subsequent experiments.

### 2.2. Pg-OMV-Induced Osteoclast Generation Is Dependent on Stimulation Timing

**Figure 2:**
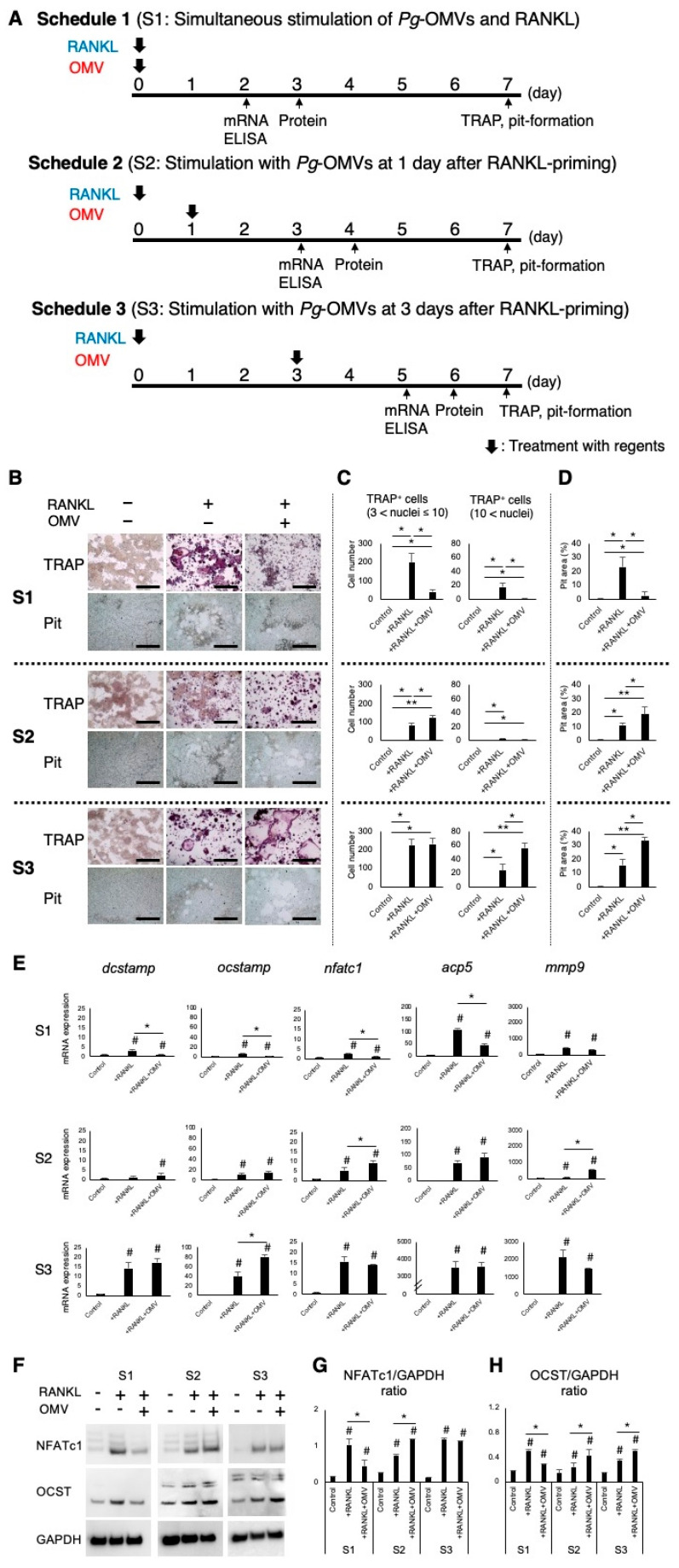
Stage-dependent effects of *Pg*-OMVs on OC-genesis. (**A**) Schematic of three stimulation schedules used in RAW264.7 cultures: Schedule 1 (S1), simultaneous stimulation with RANKL and *Pg*-OMVs at Day 0; Schedule 2 (S2), RANKL priming at Day 0 followed by *Pg*-OMVs stimulation at Day 1; and Schedule 3 (S3), RANKL priming at Day 0 followed by *Pg*-OMVs stimulation at Day 3. In all protocols, cultures were maintained for 7 days, and TRAP staining, pit-formation assays, mRNA/protein expression analyses, and cytokine analyses were performed. (**B**) Representative TRAP staining and hydroxyapatite pit resorption images under each stimulation schedule. Images of the samples were obtained using an EVOS™ XL Core Imaging System. Scale bars indicate 100 µm. (**C**) Quantification of TRAP-positive multinucleated cells (>3 nuclei and >10 nuclei). (**D**) Quantification of pit resorption area. (**E**) Relative mRNA expression of osteoclast-related markers (*dcstamp*, *ocstamp*, *nfatc1*, *acp5*, *mmp9*). (**F**–**H**) Western blot analyses of NFATc1 and OC-STAMP proteins normalized to GAPDH, with densitometric quantification. Results demonstrate that simultaneous OMV + RANKL stimulation (S1) suppressed osteoclast formation, whereas delayed *Pg*-OMVs addition (S2, S3) enhanced OC-genesis and upregulated OC-STAMP expression. The images were obtained from using the Azure C400. Results were presented as the means ± SD (n = 3). Experiments were independently repeated three times with similar results. Statistical analyses were performed using the Kruskal–Wallis test followed by Steel–Dwass’s post hoc test for multiple comparisons. #: *p* < 0.05, vs. control. *: *p* < 0.05, vs. +RANKL+*Pg*-OMVs, **: *p* ≤ 0.01, vs. +RANKL+*Pg*-OMVs.

Three experimental schedules were established based on the timing of *Pg*-OMV administration (Figure 2A). *Pg*-OMVs alone did not affect the generation of multinucleated tartrate-resistant acid phosphatase (TRAP)-positive cells (Figure S2A). Subsequently, we employed three different stimulation schedules to examine the effect of *Pg*-OMVs on macrophage/osteoclast lineage. In S1, the number of TRAP-positive cells (>3 and >10 nuclei) and resorbed hydroxyapatite area significantly decreased compared to the stimulation with RANKL alone (Figure 2A–C). Similarly, the mRNA expressions of *dendritic cell-specific transmembrane protein* (*dcstamp*), *osteoclast stimulatory transmembrane protein* (*ocstamp*), *nuclear factor of activated T-cell 1* (*nfatc1*), and *acid phosphatase 5* (*acp5*) were significantly decreased by the addition of *Pg*-OMVs co-stimulated with RANKL (Figure 2D). On the other hand, the number of TRAP-positive cells and resorbed pit area increased by the stimulation with *Pg*-OMVs in S2 (Figure 2A–C). The relative mRNA expressions of *nfatc1* and *mmp9* were significantly promoted compared to RANKL alone. In S3, the number of TRAP-positive cells with more than 10 nuclei and the resorbed pit area significantly increased. Moreover, *Pg*-OMVs significantly increased *ocstamp* mRNA expression compared with other groups. Moreover, stimulation of BMM with RANKL and *Pg*-OMVs simultaneously diminished the formation of TRAP-positive cells, whereas the addition of *Pg*-OMVs after RANKL stimulation induced increased TRAP-positive multinucleated cells (Figure S2B).

According to Western blotting analysis, in S1 group, the expressions of NFATc1 and OC-STAMP proteins in RANKL-primed RAW264.7 cells were significantly suppressed by the simultaneous stimulation with *Pg*-OMVs (Figure 2E–G and Figure S3). On the other hand, RANKL-pre-primed RAW264.7 cells increased the expression of the NFATc1 protein in response to the delayed stimulation with *Pg*-OMVs (S2; 1 day delay) (Figure 2E,F and Figure S3). The expression of the OC-STAMP protein in RANKL-pre-primed RAW264.7 cells was significantly upregulated by delayed stimulation with *Pg*-OMVs compared to RANKL-pre-priming alone in both groups of S2 and S3 (Figure 2E,G and Figure S3). In sum, expressions of the NFATc1 and OC-STAMP proteins corresponded with expression patterns of their mRNAs. These results suggest that the timing of *Pg*-OMV stimulation after RANKL priming alters the responses of RAW264.7 cells to differentiate into multinucleated osteoclasts and to resorb hydroxyapatite.

### 2.3. Blocking OC-STAMP Suppresses Pg-OMV-Mediated OC-Genesis

**Figure 3:**
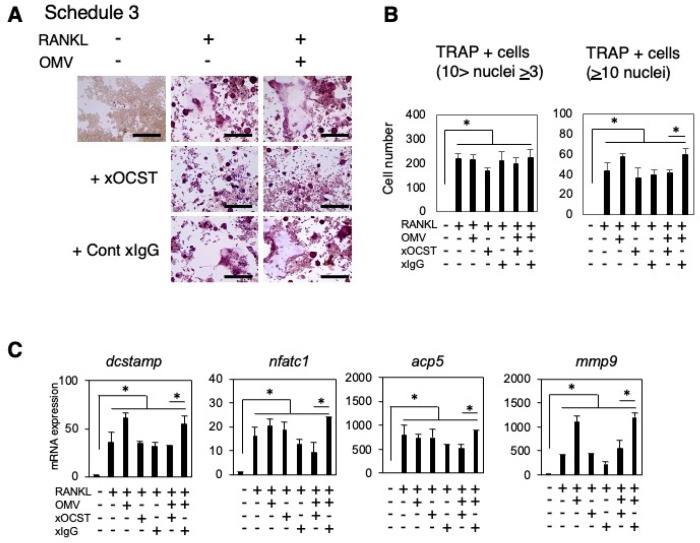
OC-STAMP mediates *Pg*-OMV-induced osteoclast fusion. (**A**) TRAP staining of RAW264.7 cells stimulated under S3 with or without neutralizing anti-OC-STAMP monoclonal antibody (xOC-STAMP mAb) or isotype IgG control. Images of the samples were obtained using an EVOS™ XL Core Imaging System. Scale bars indicate 100 µm. (**B**) Quantification of TRAP-positive multinucleated osteoclasts (>3 and >10 nuclei). (**C**) Expression of osteoclast-related genes (*dcstamp*, *nfatc1*, *acp5*, *mmp9*) by qRT-PCR. Neutralization of OC-STAMP significantly reduced *Pg*-OMV-induced osteoclast fusion and gene expression, demonstrating the role of OC-STAMP in *Pg*-OMV-mediated OC-genesis. Results were presented as the means ± SD (n = 3). Experiments were independently repeated three times with similar results. Statistical analyses were performed using the Kruskal–Wallis test followed by Steel–Dwass’s post hoc test for multiple comparisons. *: *p* < 0.05.

To evaluate the possible enrolment of OC-STAMP, an osteoclast-specific fusogen, on OC-genesis induced by *Pg*-OMVs, xOC-STAMP-mAb was used to neutralize the effect of OC-STAMP. The addition of xOC-STAMP-mAb to RAW264.7 cells that were pre-primed with RANKL and received the delayed *Pg*-OMVs significantly reduced the number of large osteoclasts with more than 10 nuclei compared to the group that received isotype control in the S3 group (Figure 3A,B) and the S2 group (Figure S4). The mRNA expression of *dcstamp*, *nfatc1*, *acp5*, and *mmp9* was also suppressed by the addition of xOC-STAMP-mAb to RAW264.7 cells that were pre-primed with RANKL and received the delayed *Pg*-OMVs compared with the groups that received isotype control (Figure 3C). These results indicate that xOC-STAMP-mAb canceled the OC-genesis upregulated by the stimulation with *Pg*-OMVs.

### 2.4. Production of Pro-Inflammatory Cytokines Was Also Altered by the Different Timings of Pg-OMVs Applied to RANKL-Primed RAW264.7 Cells

**Figure 4:**
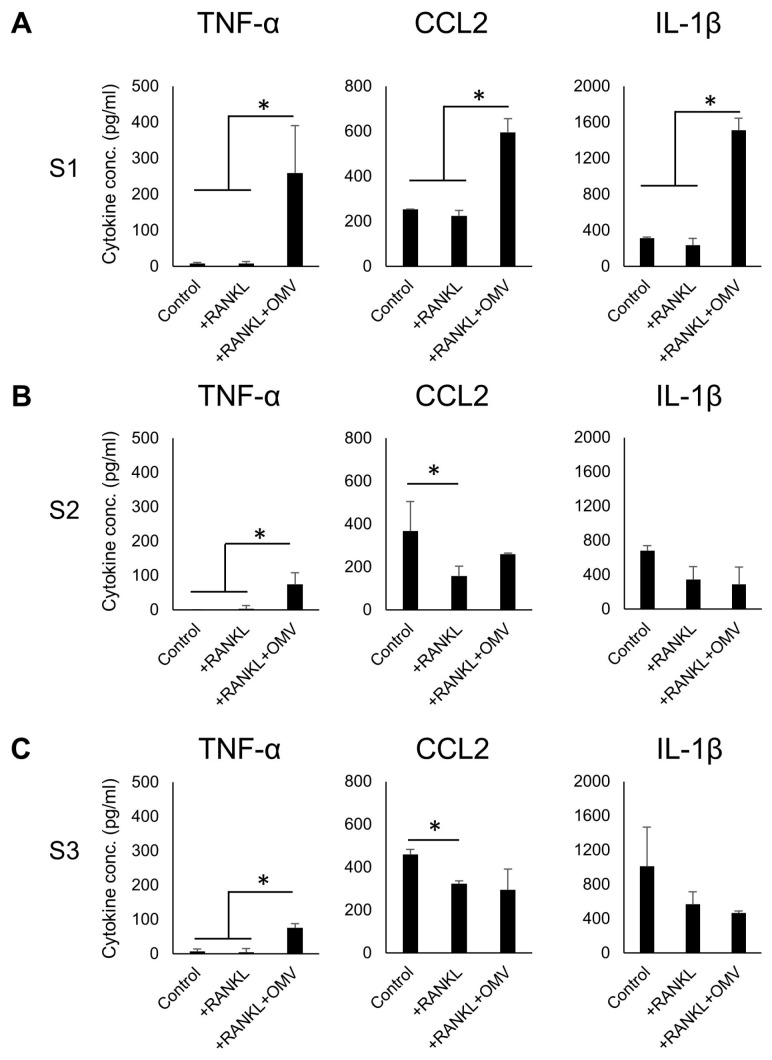
*Pg*-OMVs modulate cytokine production in RANKL-primed RAW264.7 cells. ELISA measurement of pro-inflammatory cytokines TNF-α (**A**), CCL2 (**B**), and IL-1β (**C**) in culture supernatants collected 24 h after OMV stimulation. Simultaneous stimulation (S1) markedly increased TNF-α, CCL2, and IL-1β production, while delayed stimulation (S2, S3) primarily elevated TNF-α, with lesser effects on CCL2 and IL-1β. Results were presented as the means ± SD (n = 3). Experiments were independently repeated three times with similar results. Statistical analyses were performed using the Kruskal–Wallis test followed by the Steel–Dwass post hoc test for multiple comparisons. *: *p* < 0.05.

The production of tumor necrosis factor-α (TNF-α), C-C motif chemokine ligand 2 (CCL2), and interleukin-1β (IL-1β) by RAW264.7 cells in response to stimulation with RANKL in the presence or absence of *Pg*-OMVs was measured by ELISA. After stimulating with *Pg*-OMVs, the TNF-α, CCL2, and IL-1β production from RANKL-primed RAW264.7 cells increased significantly compared to the control in the S1 group (Figure 4). In the S2 and S3 groups, while the release of TNF-α from RANKL-pre-primed RAW264.7 cells was increased by the delayed stimulation with *Pg*-OMVs, there was no significant difference in CCL2 and IL-1β productions from RANKL-pre-primed RAW264.7 cells by the delayed stimulation with *Pg*-OMVs.

### 2.5. Incidences of Macrophage Subsets After Temporally Different Stimulations of RANKL-Primed RAW264.7 Cells with Pg-OMVs

**Figure 5:**
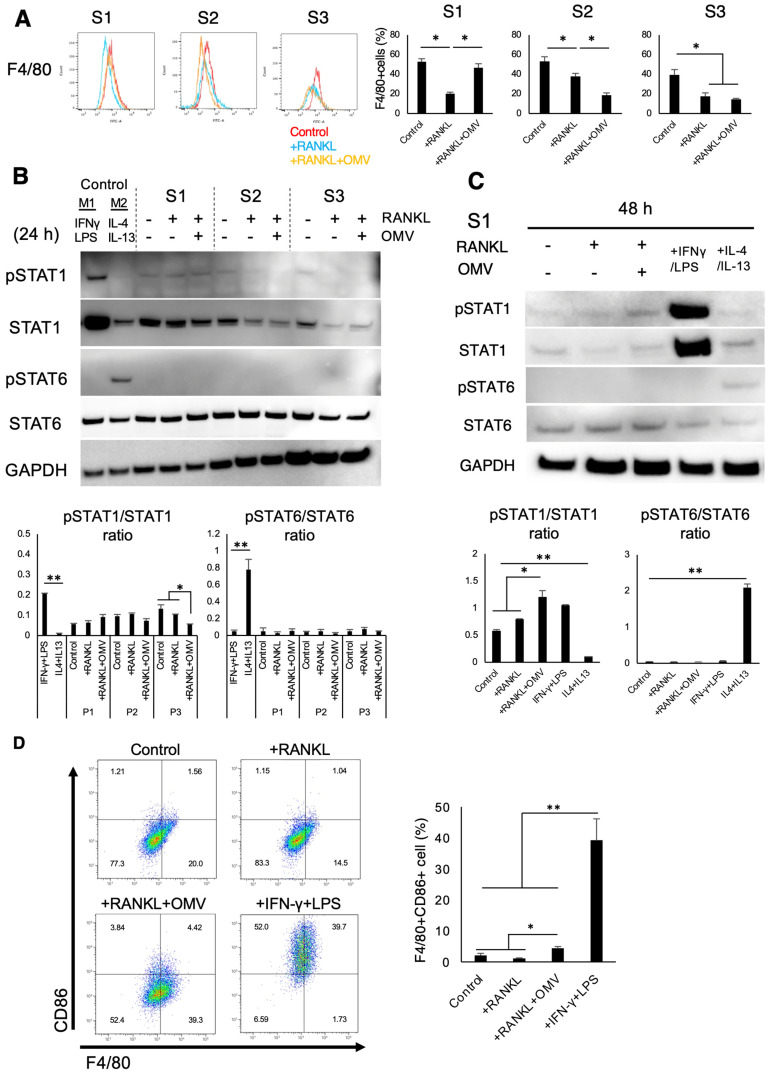
*Pg*-OMVs influence macrophage marker expression and STAT signaling. (**A**) Flow cytometry analysis of macrophage marker F4/80 expression under different stimulation schedules. *: *p* < 0.05, **: *p* < 0.05. (**B**,**C**) Western blot analysis of phosphorylated and total STAT1 and STAT6 in RAW264.7 cells after 24 h (**B**) and 48 h (**C**) stimulation. Co-stimulation (S1) modestly enhanced STAT1 phosphorylation, while delayed OMV stimulation (S2, S3) reduced STAT1 activation. STAT6 phosphorylation was not detected. The images were obtained using the Azure C400. (**D**) Flow cytometry showing increased CD86+/F4/80+ population (M1-like macrophages) in S1 compared to control. Data indicate that *Pg*-OMVs bias RANKL-primed RAW264.7 cells toward M1 polarization under early co-stimulation. The images were generated with FlowJo software (ver 11.11.1.0). Results were presented as the means ± SD (n = 3). Experiments were independently repeated three times with similar results. Statistical analyses were performed using the Kruskal–Wallis test followed by Steel–Dwass’s post hoc test for multiple comparisons. *: *p* < 0.05, **: *p* < 0.01.

The expressions of macrophage markers, i.e., F4/80 and CD11b [38,39], after stimulation of RAW264.7 cells with *Pg*-OMVs for 72 h were evaluated using a flow cytometer (Figure 5A: F4/80 and Figure S5A; CD11b). In the S1 group, the expressions of both F4/80 and CD11b were enhanced in the RANKL-primed RAW264.7 cells by simultaneous stimulation with *Pg*-OMVs, compared to RAW264.7 cells primed with RANKL alone. In S2, the expression of both macrophage markers in the RANKL-pre-primed cells that received 1-day delayed stimulation with *Pg*-OMVs was lower than that of Raw264.7 cells that received RANKL-pre-priming alone or in control (no stimulation). However, there was no difference in F4/80 and CD11b expression on RANKL-pre-primed RAW264.7 cells between 3-day delayed stimulation with *Pg*-OMVs and RANKL-pre-priming alone in the S3 group.

It is well established that signal transducers and activators of transcription (STAT)-family transcription factors are engaged in macrophage polarization toward M1 or M2 subsets [40]. STAT1 activation promotes M1 macrophage polarization [41], while Stat6 drives macrophage M2 polarization [42]. To evaluate the possible effects of *Pg*-OMVs on macrophage polarization, Western blotting was performed to determine the phosphorylation level of STAT proteins in the cell lysate collected from RANKL-primed RAW264.7 cells stimulated with *Pg*-OMVs for 24 h (Figure S5B: detailed stimulation and sampling timelines in S1, S2 and S3 groups). There was no statistical difference in the phosphorylated(p)-STAT1/total(t)-STAT1 ratio between RANKL-primed RAW264.7 cells without *Pg*-OMVs and those stimulated with *Pg*-OMVs in S1 as well as S2. In contrast, the pSTAT1/tSTAT1 ratio significantly decreased in S3 compared to the other groups (Figure 5B and Figure S6). It is noteworthy that the S1 group showed a trend of increase in pSTAT1/tSTAT1 as compared to *Pg*-OMVs/RANKL-stimulated RAW274.7 cells to those stimulated (primed) with RANKL alone. STAT6 phosphorylation was not detected in any groups tested (S1, S2, and S3) (Figure 5B and Figure S6). However, in the S1 group, after 48 h (Figure 5C and Figure S7) and 72 h (Figures S5C and Figure S7) from the stimulation of RAW264.7 cells with both RANKL and *Pg*-OMVs simultaneously, the pSTAT1/tSTAT1 ratio was statistically enhanced compared to RAW264.7 cells primed with RANKL alone (Figure 5C and Figure S7), suggesting that M1-like phenotypes were induced in S1 by stimulation with *Pg*-OMVs for more than 48 h. In all three groups, S1, S2, and S3, the non-detectable level of the pSTAT6/tSTAT6 ratio did not change by the stimulation with *Pg*-OMVs via the incubation of RAW264.7 cells for 48 h (Figure 5C and Figure S7). According to flow cytometry results, there was a slightly greater percentage of the enhanced M1 macrophage markers CD86 and F4/80 with co-stimulation (4.42% in quadrant 2) when compared to the control (1.01%) (Figure 5D). These results indicated that a small population (about 5%) of RAW264.7 cells was polarized to M1-like macrophages as they were stimulated with RANKL and *Pg*-OMVs more than 48 h.

### 2.6. Pg-OMVs’ Effect on Phagocytotic Activity of RANKL-Primed RAW264.7 Cells

**Figure 6:**
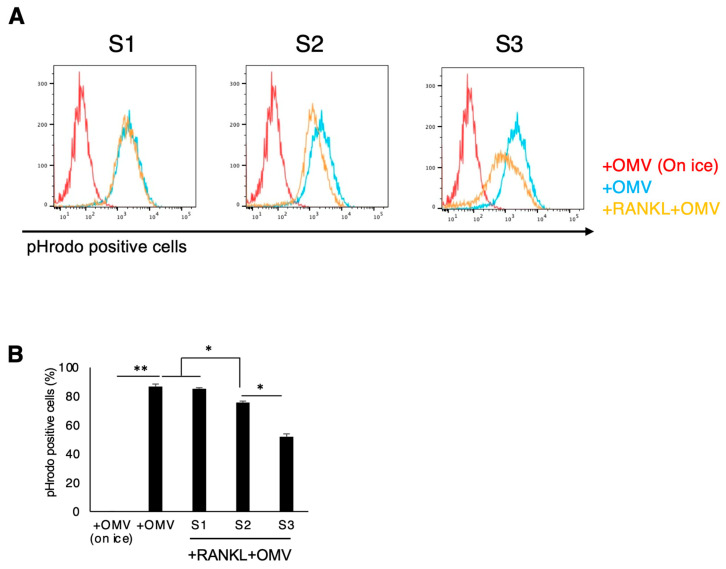
Phagocytosis of *Pg*-OMVs by RANKL-primed RAW264.7 cells. (**A**) Flow cytometry analysis of *Pg*-OMV uptake using pHrodo-labeled OMVs under three stimulation schedules. The images were generated with FlowJo software (ver 11.11.1.0). (**B**) Quantification of pHrodo-positive cells shows significantly lower OMV uptake in RANKL-pre-primed cells (S2 and S3) compared to co-stimulation (S1). The reduced phagocytosis was negatively correlated with enhanced osteoclast differentiation in S2 and S3. Results were presented as the means ± SD (n = 3). Experiments were independently repeated three times with similar results. Statistical analyses were performed using the Kruskal–Wallis test followed by Steel–Dwass’s post hoc test for multiple comparisons. *: *p* < 0.05, **: *p* < 0.01.

The phagocytosis of exogenous microorganisms is equipped in both M1 and M2 macrophages [43]. Important to this study, Lam et al. reported that M1 and M2 macrophages exhibit an enhanced capacity for *Pg* phagocytosis compared with that of naive macrophages [44]. There was no significant difference in pHrodo-positive cells, which indicate cells engulfing *Pg*-OMVs, in S1, while priming with RANKL decreased pHrodo-positive cells in S2 and S3. In comparison with S1, there was a smaller number of pHrodo-positive cells in S2 and S3 (Figure 6A,B).

### 2.7. Pg-OMVs Enhance RAW264.7 Cells’ Migration

**Figure 7:**
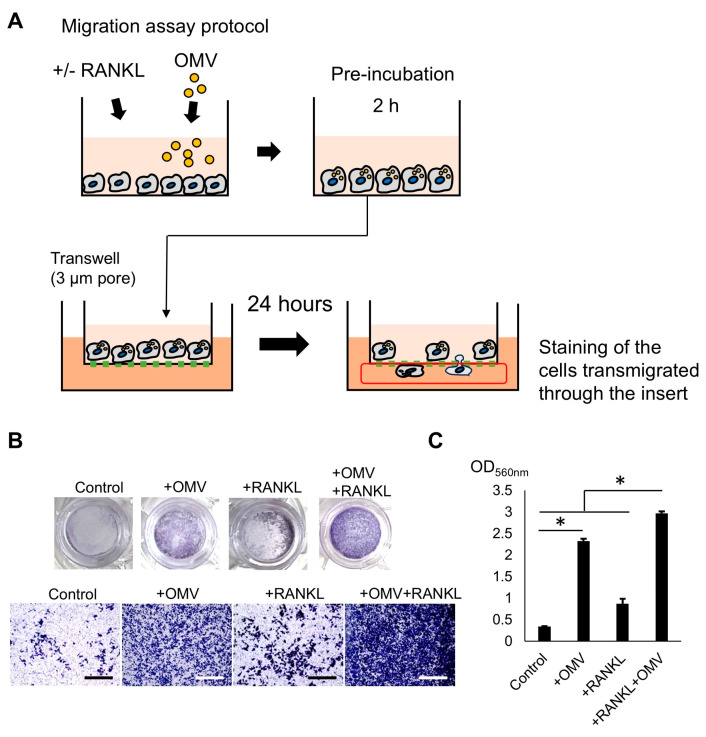
*Pg*-OMVs enhance RAW264.7 cell migration. (**A**) Schematic of Transwell migration assay: RAW264.7 cells pretreated with RANKL and/or *Pg*-OMVs were seeded in the upper chamber, with migration induced by FBS gradient. (**B**) Representative crystal violet staining of migrated cells across polycarbonate membranes. Images from the samples were obtained via EVOS™ XL Core Imaging System. Scale bars indicate 100 µm. (**C**) Quantitative absorbance (OD560 nm) of eluted dye from migrated cells. Simultaneous OMV + RANKL stimulation (S1) significantly enhanced cell migration compared to RANKL alone, indicating a role of *Pg*-OMVs in promoting macrophage/osteoclast precursor motility. Results were presented as the means ± SD (n = 3). Experiments were independently repeated three times with similar results. Statistical analyses were performed using the Kruskal–Wallis test followed by Steel–Dwass’s post hoc test for multiple comparisons. *: *p* < 0.05.

Evaluating the cell migration caused by *Pg*-OMV priming, when stimulating the RAW264.7 cells with *Pg*-OMVs, there was significantly greater migration across the polycarbonate membrane toward the increased serum (fetal bovine serum, FBS) gradient. Additionally, the co-stimulation of RANKL and *Pg*-OMVs further enhanced their migratory function, resulting in greater migration (Figure 7B,C).

## 3. Discussion

Periodontal disease is characterized by progressive destruction of periodontal tissue and alveolar bone loss [1,45]. The imbalance between host immune response and oral bacterial infection plays a vital role in disease progression, with *Pg* considered a keystone pathogen among periodontitis-associated microbes [10,11]. *Pg* produces abundant OMVs [12], which, owing to their permeability, can exacerbate inflammation [46] and promote tissue destruction [47]. However, evidence regarding their specific effects on host–pathogen interactions remains limited.

In this study, we demonstrated the effect of *Pg*-OMVs on macrophage/osteoclast differentiation from monocytes using the RAW264.7 monocyte cell line. We first confirmed the diameter of *Pg*-OMVs. Purified *Pg*-OMVs were 241 nm on average, as previously reported [12]. A cell proliferation assay showed that *Pg*-OMVs were not cytotoxic at concentrations ranging from 0.1 to 50 µg/mL. For this study, we used *Pg*-OMVs at 1 µg/mL, a concentration that elicited the strongest proliferative response without inducing cytotoxicity in RAW264.7 cells. Several previous studies analyzing *Pg*-OMV-mediated cellular responses have also used 1 µg/mL *Pg*-OMVs [48,49]. We used a single concentration of *Pg*-OMVs to uniformly evaluate their various biological activities on macrophages and osteoclasts, including effects on osteoclast differentiation, inflammatory cytokine production, phagocytosis, and migration. However, conducting experiments using multiple concentrations in future studies would provide additional valuable insights.

To investigate the effect of *Pg*-OMVs on macrophage/osteoclast linage, we employed three distinct stimulation schedules that captured key developmental phases of OC-genesis. These schedules allowed us to evaluate *Pg*-OMVs exposure prior to RANKL stimulation (S1), during early precursor differentiation (S2), and during late differentiation and maturation (S3). While *Pg*-OMVs and RANKL were added to RAW264.7 cells at the same time in S1, cells were first primed with RANKL to induce osteoclast precursor differentiation before *Pg*-OMV stimulation in S2 and S3. Although the duration of *Pg*-OMV exposure differs among the three schedules, this design was intentional. Prolonged exposure, particularly in S2 and S3, where osteoclast differentiation progresses, can lead to increased cell death and obscure stage-dependent differences. Therefore, the exposure periods were adjusted to ensure that the timing-specific effects of *Pg*-OMVs could be accurately evaluated. As a result, we observed that temporally different effects of *Pg*-OMVs, depending on the timing of *Pg*-OMV treatment. Early-stage exposure to *Pg*-OMVs suppressed osteoclast generation while promoting macrophage differentiation. In contrast, late-stage treatment with *Pg*-OMVs enhanced osteoclast formation without supporting macrophage differentiation. These findings suggest that *Pg*-OMVs play a decisive role in directing macrophage fate, influencing whether cells commit to macrophage maintenance or osteoclast differentiation.

Firstly, stimulation of *Pg*-OMVs after RANKL priming promoted osteoclast differentiation. In S2, *Pg*-OMVs significantly increased the expression of NFATc1—the master regulator of OC-genesis [50]—at both the gene and protein levels compared with RANKL alone. This indicates that *Pg*-OMVs enhance the early phase of osteoclast differentiation. Furthermore, *Pg*-OMV stimulation following prolonged RANKL priming (S3) did not further NFATc1 expression relative to RANKL alone; however, it notably promoted the formation of multinucleated osteoclasts and upregulated OC-STAMP expression. OC-STAMP is known as a cell fusion protein which involves the formation of multinucleated osteoclasts [51]. These findings suggest that *Pg*-OMVs may particularly facilitate cell fusion during the later stages of differentiation and thereby contribute to osteoclast maturation. OC-STAMP, a six-transmembrane protein with three extracellular loops, is distinctively expressed by osteoclast precursors and mediates cell fusion to form multinucleated osteoclasts [51]. In previous studies, OC-STAMP knockout mice exhibited no abnormal bone phenotype [52]. In contrast, OC-STAMP knockout mice exhibited no abnormal bone phenotype under homeostatic conditions [53], suggesting a pathogenic rather than homeostatic role of OC-STAMP in bone remodeling. To clarify the relationship between *Pg*-OMVs and OC-STAMP, we employed a monoclonal antibody against OC-STAMP. Treatment with this antibody significantly suppressed the formation of multinucleated osteoclasts compared with an isotype-matched control. These results indicate that *Pg*-OMVs may enhance OC-STAMP expression to promote osteoclast fusion, thereby driving pathogenic bone resorption. Although additional studies are needed to fully elucidate the underlying mechanism, our findings suggest that anti-OC-STAMP monoclonal antibody can represent a promising candidate for preventing *Pg*-OMV-induced pathological bone loss. Previous studies [53] showing that a monoclonal antibody against OC-STAMP suppresses silk ligature-induced alveolar bone resorption also support this hypothesis.

In contrast, when *Pg*-OMVs were stimulated simultaneously with RANKL (S1), osteoclast differentiation was suppressed. In summary, both mRNA and Western blotting analyses demonstrated that *Pg*-OMVs downregulated NFATc1 expression in RAW264.7 cells co-stimulated with RANKL, whereas adding *Pg*-OMVs to RANKL-primed osteoclast precursors enhanced osteoclast differentiation, increased osteoclast numbers, and elevated the expression of osteoclast-related genes, including NFATc1. The biphasic effects of *Pg*-OMVs on OC-genesis observed in this study may be attributed to pathogenic components contained within the OMVs. *Pg*-OMVs are known to contain multiple virulence factors such as LPS and gingipain [17,54]. Previous study has shown that LPS inhibits RANKL-mediated OC-genesis at early stages by modulating NFATc1 expression but enhances OC-genesis in later stages through Toll-like receptor 4 (TLR4)-dependent mechanisms [55]. These findings support the possibility that the biphasic effects of *Pg*-OMVs on osteoclast differentiation may be mediated, at least in part, by their LPS content. Furthermore, Kim et al. reported that *Pg*-OMV-associated LPS induces osteoclast generation activity in RANKL-pretreated precursors via TLR2 [30], which is consistent with our results. In addition, a culture supernatant derived from live *Pg* cells was shown to promote OC-genesis, and this effect was attenuated by polymyxin B, indicating an LPS-dependent mechanism [56]. Thus, *Pg*-OMVs may induce osteoclast differentiation via LPS-TLR signaling; however, which TLRs are involved and the downstream pathways remain to be elucidated. Other *Pg*-derived components may also contribute to these effects, as gingipains have been reported to enhance OC-genesis in RAW264.7 cells [57], and *Pg*-derived ceramides have been shown to promote OC-genesis [58]. Identifying the specific OMV-associated factors responsible for regulating osteoclast differentiation will require further investigation.

Additionally, when *Pg*-OMVs were added simultaneously with RANKL (S1), the macrophage markers F4/80 or CD11b remained expressed, suggesting that *Pg*-OMVs suppress RANKL-driven OC-genesis by maintaining a macrophage-like phenotype. We therefore investigated the impact of *Pg*-OMVs on macrophage activation. In S1, *Pg*-OMVs increased the production of inflammatory cytokines, including TNF-α, CCL2, and IL-1β. *Pg*-OMVs have been reported to induce TNF-α and IL-1β production by macrophages [31,46], and *Pg*-derived LPS, which is contained abundantly in *Pg*-OMVs, is known to induce CCL2 production [59,60]. Consistent with these reports, our results indicate that, under S1 conditions, *Pg*-OMVs drive macrophage differentiation rather than OC-genesis. Although CCL2 and IL-1β production were suppressed by RANKL stimulation, *Pg*-OMV-induced TNF-α release was observed in S2 and S3. Because TNF-α promotes osteoclast differentiation [61], *Pg*-OMV-induced TNF-α production may enhance OC-genesis in these conditions.

Our results further showed that *Pg*-OMVs weakly promoted the phosphorylation of STAT1 without markedly affecting the expression of macrophage markers such as F4/80. STAT signaling is a key regulator of macrophage polarization [40]; STAT1 has an essential activity for macrophage polarization toward M1 [41], whereas STAT6 is responsible for M2 polarization [42]. According to immunoblotting, in S2 and S3, the phosphorylation of STAT1 was suppressed by *Pg*-OMVs, consistent with enhanced OC-genesis under these conditions. In S1, *Pg*-OMVs did not affect the phosphorylation of STAT1 or STAT6 at 24 h; however, by 48 h, STAT1 phosphorylation was weakly promoted, accompanied by a modest rise in F4/80- and CD86-positive cells. These results indicated that *Pg*-OMVs induce mild M1 polarization even in the presence of RANKL, potentially suppressing OC-genesis. This aligns with previous reports showing that *Pg*-LPS weakly activated M1 and M2 polarization and strongly induced inflammatory cytokines [62] and that *Pg*-OMVs harvested at the late log or stationary phase preferentially promote M1 polarization [63]. Collectively, these results indicate that *Pg*-OMVs, when added simultaneously with RANKL, may bias macrophages toward an “M1-like” phenotype and promote inflammatory activation, thereby suppressing osteoclast differentiation. However, further studies using additional macrophage markers will be required to clarify this point fully.

Recent advances in single-cell transcriptomics have revealed that OC-genesis is neither a linear nor a uniformly successful process. Tsukasaki et al. identified a distinct subpopulation of precursor cells that fail to complete the differentiation program toward mature multinucleated osteoclasts, which they termed “failed osteoclasts” [64]. Their biological significance, whether they represent passive by-products or active participants in inflammatory bone disorders, remains incompletely understood. Our findings suggest that *Pg*-OMVs may actively generate such a population of “failed osteoclasts”. As described above, *Pg*-OMVs suppress osteoclast differentiation even in the presence of RANKL, and we demonstrated that these cells internalize *Pg*-OMVs. This observation aligns with the report by Nishimura et al., showing that TRAP-positive osteoclast precursors fail to differentiate into mature osteoclasts upon continuous stimulation with RANKL after *Escherichia coli* phagocytosis [33]. Furthermore, *Pg*-OMV-phagocytosing cells exhibited enhanced migratory activity upon RANKL stimulation. This suggests that these “dysfunctional osteoclast-like” cells do not simply become inert but instead acquire new functional properties, potentially acting as mobile carriers/quenchers of *Pg*-OMVs within inflamed periodontal tissues. To fully elucidate the pathological relevance of this cell population, further studies incorporating lineage tracing, single-cell analyses, and functional assays will be essential.

We demonstrated that *Pg*-OMVs exert stage-dependent, dual effects on the monocyte–macrophage–osteoclast lineage. Given that local injection of *Pg*-OMVs reportedly promoted alveolar bone destruction in mice [63,65], *Pg*-OMVs can eventually enhance the bone resorption. Our findings suggest that *Pg*-OMV exposure during early monocyte differentiation heightens inflammation, whereas exposure of osteoclast precursors promotes OC-genesis, together driving periodontal disease progression. The biphasic effects of *Pg*-OMVs on OC-genesis, suppressing early differentiation while promoting late-stage maturation and cell fusion, may have important translational implications for periodontal disease pathogenesis. In inflamed periodontal tissues, macrophages and osteoclast precursors are continuously exposed to fluctuating levels of bacterial products, including OMVs released by *Pg*. Our findings suggest that *Pg*-OMVs may initially maintain macrophages in an undifferentiated state, potentially prolonging their inflammatory activity. However, once precursors are primed by RANKL within the osteolytic microenvironment, *Pg*-OMVs may accelerate their transition toward multinucleated, bone-resorbing osteoclasts. This stage-specific modulation provides a potential mechanistic explanation for the rapid and localized bone destruction observed in periodontitis lesions, where bacterial signals and RANKL-rich inflammatory cues coexist. Understanding this temporal regulation of OC-genesis by *Pg*-OMVs may inform the development of therapeutic strategies to disrupt late-stage osteoclast maturation or OMV-mediated signaling in order to prevent excessive alveolar bone resorption. In addition, *Pg*-OMVs are relevant to distant tissue dysfunction, including cardiovascular, brain, and joint disorder [66]. Macrophages that internalize *Pg*-OMVs may fail to complete osteoclast differentiation and instead disseminate vesicle-borne virulence factors. Collectively, these findings suggest a mechanistic link between *Pg*-OMVs and pathological bone resorption in periodontitis. However, since *Pg*-OMVs has various virulence factors, further studies are needed to identify the specific effectors that modulate the macrophage/osteoclast lineage.

This study has several limitations. First, although potential mediators such as OC-STAMP and TLR-associated pathways have been proposed to explain the biphasic effects of *Pg*-OMVs on osteoclast differentiation, the precise molecular mechanisms remain to be fully elucidated. Moreover, while our study primarily utilized RAW264.7 cells to evaluate the impacts of *Pg*-OMVs on OC-genesis and macrophage responses, we acknowledge the limitations of relying on this immortalized cell line as a substitute for primary osteoclast precursors. RAW264.7 cells provide a convenient and reproducible model; however, they differ from BMMCs in several aspects, including their responsiveness to RANKL, fusion capacity, and expression dynamics of OC-related markers [67,68]. These differences may influence the magnitude or timing of *Pg*-OMV-induced effects observed in our assays. To partially address this limitation, we validated the formation of multinucleated osteoclast formation using BMMCs. However, future studies incorporating a broader set of primary cell-based functional assays will be essential to more fully elucidate how *Pg*-OMVs modulate osteoclast differentiation and function in physiological contexts. Finally, the clinical relevance of *Pg*-OMV-mediated modulation of the osteoclast lineage requires further investigation using an appropriate in vivo model, including a *Pg*-OMV-induced periodontitis model combined with OC-STAMP-neutralizing antibody or osteoclast precursor-specific TLR knockout mice.

## 4. Materials and Methods

### 4.1. Preparation of Pg-OMVs

OMVs were isolated from *Pg*W83 (BAA-308, ATCC, Manassas, VA, USA) using a method described previously [19,20]. Briefly, *Pg* was cultured on a blood agar plate (Culture Media & Supplies Inc., Oswego, IL, USA) at 37 °C in anaerobic conditions by AnaeroPack™-Anaero Anaerobic Gas Generator (MITSUBISHI Gas Chemical, Tokyo, Japan). A single *Pg* colony was selected and inoculated into tryptic soy broth supplemented with 5 µg/mL hemin, 2 mg/mL menadione, and 5 mM L-cystine HCl for 7 days at 37 °C. To isolate *Pg*-OMVs, 30 mL of the bacterial supernatant was collected and centrifuged at 4 °C, 8000× *g*, for 20 min (Avanti J-E Centrifuge, Beckman Coulter, Miami, FL, USA). The supernatant was collected and filtered through a 0.22 µm filter (MilliporeSigma, Burlington, MA, USA). The samples, passed through a 0.22 µm filter, were ultracentrifuged at 4 °C, 100,000× *g*, for 1 h (Optima XPN-80, Beckman Coulter, Brea, CA, USA). The resulting pellet was dissolved in PBS, and its protein concentration was measured by BCA assay (Thermo Fisher Scientific, Waltham, MA, USA). The size distribution was characterized using the NanoSight NS300 Instrument (Malvern Panalytical, Worcestershire, UK) to confirm the presence of nanosized *Pg*-OMVs.

### 4.2. Cell Culture and Osteoclast Differentiation

RAW264.7 cells (ATCC, TIB-71^TM^, Manassas, VA, USA), a murine macrophage-like cell line, were cultured in Dulbecco’s Modified Eagle Medium (DMEM, Thermo Fisher Scientific) supplemented with 10% FBS at 37 °C, 5% CO_2_, and 95% humidity. Cells were cultured in wells of a 96-well culture plate (Corning Inc., Corning, NY, USA) to determine the optimal concentration by a water-soluble tetrazolium dye-8 (WST-8) assay (Abcam, Cambridge, MA, USA). For osteoclast differentiation assay, RAW264.7 cells were plated in wells of a 96-well culture plate at a density of 3 × 10^3^ cells/well. Three schedules were employed to induce osteoclast differentiation from RAW264.7 cells primed with 10 ng/mL of RANKL (BioLegend, San Diego, CA, USA) (Figure 2A). The concentration of RANKL was determined based on a previous study [69]. Briefly, in Schedule 1 (S1), RAW264.7 cells that received RANKL with or without *Pg*-OMVs at Day-0 were incubated for 7 days. In Schedule 2 (S2) or Schedule 3 (S3), RAW264.7 cells were pre-primed with RANKL alone for 24 (S2) or 72 h (S3), followed by the stimulation with *Pg*-OMVs in the absence of RANKL for a total of 7 days, respectively. Differentiated mature osteoclasts were identified by tartrate-resistant acid phosphatase (TRAP) staining (Sigma-Aldrich, St. Louis, MO, USA). TRAP-positive cells with ≥3 or 10 nuclei were counted as mature osteoclasts. Moreover, a pit-formation assay using hydroxyapatite (HA)-coated 96-well plates was conducted [70]. After inducing the differentiation into osteoclasts, cells were removed with 10% bleach. Images from the samples were obtained via EVOS™ XL Core Imaging System (Thermo Fisher Scientific) and pit areas were analyzed with a public domain software (ImageJ, ver. 2.1.0/1.53c, National Institutes of Health, Bethesda, MD, USA). Moreover, bone marrow-derived mononuclear cells (BMMC, frozen cells, BALB-5030) derived from BALB/c male mice were obtained from Cell Biologics (Chicago, IL, USA). To evaluate OC-genesis from BMMCs, two different schedules were employed (Figure S1B). Schedule 4 (S4): BMMCs were simultaneously stimulated with M-CSF (25 ng/mL, BioLegend, San Diego, CA, USA) and RANKL (10 ng/mL), with or without *Pg*-OMVs (1 µg/mL). Schedule 5 (S5): BMMCs were first primed with M-CSF and RANKL for 24 h and then stimulated with M-CSF and RANKL, with or without *Pg*-OMVs, for an additional 24 h (concentrations of all reagents are the same as those used in S4). The concentration of M-CSF and RANKL was determined based on a previous study [71]. The differentiated mature osteoclasts were evaluated by counting TRAP-positive cells.

### 4.3. Quantitative Real-Time PCR

RAW264.7 cells plated in wells of a 12-well culture plate at a density of 3.5 × 10^4^ cells/mL were treated following the above-noted three schedules. Using PureLink™ RNA Mini Kit (Invitrogen™, Thermo Fisher Scientific, Waltham, MA, USA), mRNA was isolated from the samples. cDNA was synthesized using the VERSO cDNA kit (Thermo Fisher Scientific, MA, USA). A PCR reaction was carried out at 95 °C for 20 s, followed by 40 cycles at 95 °C for 1 s and 4 cycles at 60 °C for 20 s, in accordance with a previous study [69]. The mRNA levels of the target genes were monitored relative to each other and were normalized to GAPDH as an internal control gene. The gene expressions of *ocstamp* (Mm00512445_m1), *dcstamp* (Mm04209236_m1), *nfatc1* (Mm01265944_m1), *acp5* (Mm00475698_m1), and *mmp9* (Mm00442991_m1) were examined. All taqman probes were obtained from Thermo Fisher Scientific.

### 4.4. Anti-OC-STAMP Antibody Preparation and Assessment of Osteoclast Generation

Mouse monoclonal anti-OC-STAMP antibody (xOC-STAMP mAb) was developed by using the conventional hybridoma technique [53]. The monoclonal antibody was obtained from a hybridoma cell line established previously in our laboratory (originally described in [53]). The hybridoma was generated under approved animal experimentation procedures, and no animals were used in the present study. As for the method, briefly, we immunized 8-week-old BALB/c mice subcutaneously with OC-STAMP peptide (FASMQRSFQWELRFTPHDCHLPQAQPPR), which was designed using a BLAST search (ver. 2.8.1, National Institutes of Health, Bethesda, MD, USA). Four weeks after primary immunization, we boosted the mice and then collected the spleens. Spleen cells were fused with SP2 myeloma cell line, and then hybridoma cells were cloned by limiting dilution. Monoclonal anti-OC-STAMP antibody (IgG3) produced by hybridoma cells was purified with a Protein A/G column (Thermo Fisher Scientific, Waltham, MA, USA). Purified anti-OC-STAMP antibody (20 µg/mL) was applied to RAW264.7 cells at the same time as adding *Pg*-OMVs in S2 and S3. Isotype-matched IgG was used as a negative control. The osteoclast generation was evaluated with TRAP staining and quantitative real-time PCR.

### 4.5. Western Blotting

Western blotting was conducted with reference to previous reports [69]. RAW264.7 cells were cultured in the three different schedules and were lysed in the wells of a 12-well cell culture plate using M-PER™ Mammalian Protein Extraction Reagent (Thermo Fisher Scientific) supplemented with Halt Protease Inhibitor Cocktails diluted 100× (Thermo Fisher Scientific). The proteins separated in the polyacrylamide gel electrophoresis (SDS-PAGE) gel were trans-blotted to a poly vinylidene di-fluoride (PVDF) membrane. The following antibodies were applied as primary antibodies: rabbit anti-GAPDH (#5174, Cell Signaling Technology, Beverly, MA, USA), mouse anti-NFATc1 (sc-7294, Santa Cruz Biotechnology, Santa Cruz, CA, USA), mouse anti-OCSTAMP, rabbit anti-pSTAT1 (#7649, Cell Signaling Technology), rabbit anti-pSTAT6 (Cell Signaling Technology), rabbit anti-STAT1 (#9172, Cell Signaling Technology), and rabbit anti-STAT6 (Cell Signaling Technology). We used HRP-labeled anti-rabbit or anti-mouse antibody (Cell Signaling Technology) as a secondary antibody. Peroxidase substrate for enhanced chemiluminescence (ECL, Pierce™, Thermo Fisher Scientific, Waltham, MA, USA) was used to develop a chemiluminescence reaction specific to the antibodies bound to the PVDF membranes using the Azure C400 (Azure Biosystems Inc., Dublin, CA, USA).

### 4.6. ELISA

RAW264.7 cells were cultured in 3 different schedules. The supernatant was collected 24 h after stimulation with *Pg*-OMVs for use in enzyme-linked immunosorbent assay (ELISA). The released production of TNF-α (Mouse TNF-alpha DuoSet ELISA, BioLegend, CA, USA), CCL2 (Mouse CCL2/JE/MCP-1 DuoSet ELISA, BioLegend, San Diego, CA, USA), and IL-1β (Mouse IL-1β DuoSet ELISA, BioLegend) was quantified.

### 4.7. Flow Cytometry

Flow cytometry was performed with reference to a previous report [72]. After stimulation period of the three protocols, the RAW264.7 cells were stained with Zombie Violet (BioLegend, CA, USA) to gate live cells and then stained with FITC-conjugated anti-F4/80 (BioLegend, CA, USA) or anti-CD11b (BioLegend, CA, USA) antibodies. To evaluate the differentiation into M1 macrophage in S1, APC-conjugated anti-CD86 antibody (BioLegend, CA, USA) was used. The live cells were gated, and the positive cells were quantified by flow cytometry (Figure S8) (BD LSRFortessa X-20 Cell Analyzer, BD Biosciences, Franklin Lakes, NJ, USA). The results were analyzed using FlowJo software (ver 11.11.1.0, BD Biosciences, NJ, USA).

### 4.8. Phagocytosis Assay

The phagocytosis assay was performed using a method described previously [46]. *Pg*-OMVs were labeled with pHrodo^TM^ Red (Thermo Fisher Scientific, Waltham, MA, USA) according to the manufacturer’s instructions to evaluate phagocytosis using flow cytometry. After washing pHrodo-labeled *Pg*-OMVs with DMEM and preparing RAW264.7 cells in the well of a 6-well plate, the cells were stimulated at each schedule. Cells were incubated on ice to be used as a negative control. After 2 h, cells were harvested and analyzed with flow cytometry (BD Biosciences, Franklin Lakes, NJ, USA). Results were analyzed using the FlowJo software (BD Biosciences).

### 4.9. Migration Assay

To investigate the characteristics of cells stimulated with RANKL and *Pg*-OMVs in S1, cell migration assay was employed (Figure 7A) following the protocol published by others [73,74]. Briefly, RAW264.7 cells were stimulated with *Pg*-OMVs in wells of a 6-well plate for 2 h in the presence or absence of 10 ng/mL RANKL and transferred into transwell migration plates (Thermo Fisher Scientific) (Figure 7A, Scheme). Cell migration was induced by 10% FBS that was applied to the bottom compartment. In 24 h, migratory cells passing through the polycarbonate membrane were evaluated by staining with 1% crystal violet solution (Sigma-Aldrich, MO, USA). Stained cells were eluted with 75% ethanol and 2% HCl from the membrane, followed by measuring absorbance at 560 nm.

### 4.10. Statistical Analysis

All quantitative data are represented as means ± SD. To conduct the comparison among datasets, the Kruskal–Wallis test followed by a Steel–Dwass (multiple comparisons) or Steel (comparison with control) post hoc test was performed. Sample sizes were determined based on pilot experiments to ensure sufficient statistical power to detect biologically meaningful differences. Each statistical analysis was carried out using EZR software (ver. 1.68, Saitama Medical Center, Jichi Medical University, Saitama, Japan) [75]. A *p* value of <0.05 was regarded as statistically significant.

## 5. Conclusions

*Pg*-OMVs exert stage-dependent, bidirectional effects on the macrophage–osteoclast lineage. They suppress OC-genesis during early differentiation, likely by maintaining macrophage identity and enhancing inflammatory activation, but enhance osteoclast formation at later stages by upregulating osteoclast-related molecules, including OC-STAMP and fusion activity.

## Data Availability

The original contributions presented in this study are included in the article/Supplementary Material. Further inquiries can be directed to the corresponding authors.

## Supplementary Materials

The following supporting information can be downloaded at: https://www.mdpi.com/article/10.3390/ijms27020831/s1.
