# Supplementary material for: Porphyromonas gingivalis Vesicles Control Osteoclast–Macrophage Lineage Fate
Source: Int J Mol Sci. 2026 Jan 14;27(2):831. doi: 10.3390/ijms27020831 (PMC12840716; doi:10.3390/ijms27020831)
Supplement: Supplementary file 1 [file ijms-27-00831-s001.zip › ijms-3974407-supplementary.pdf]

Supplementary Figures

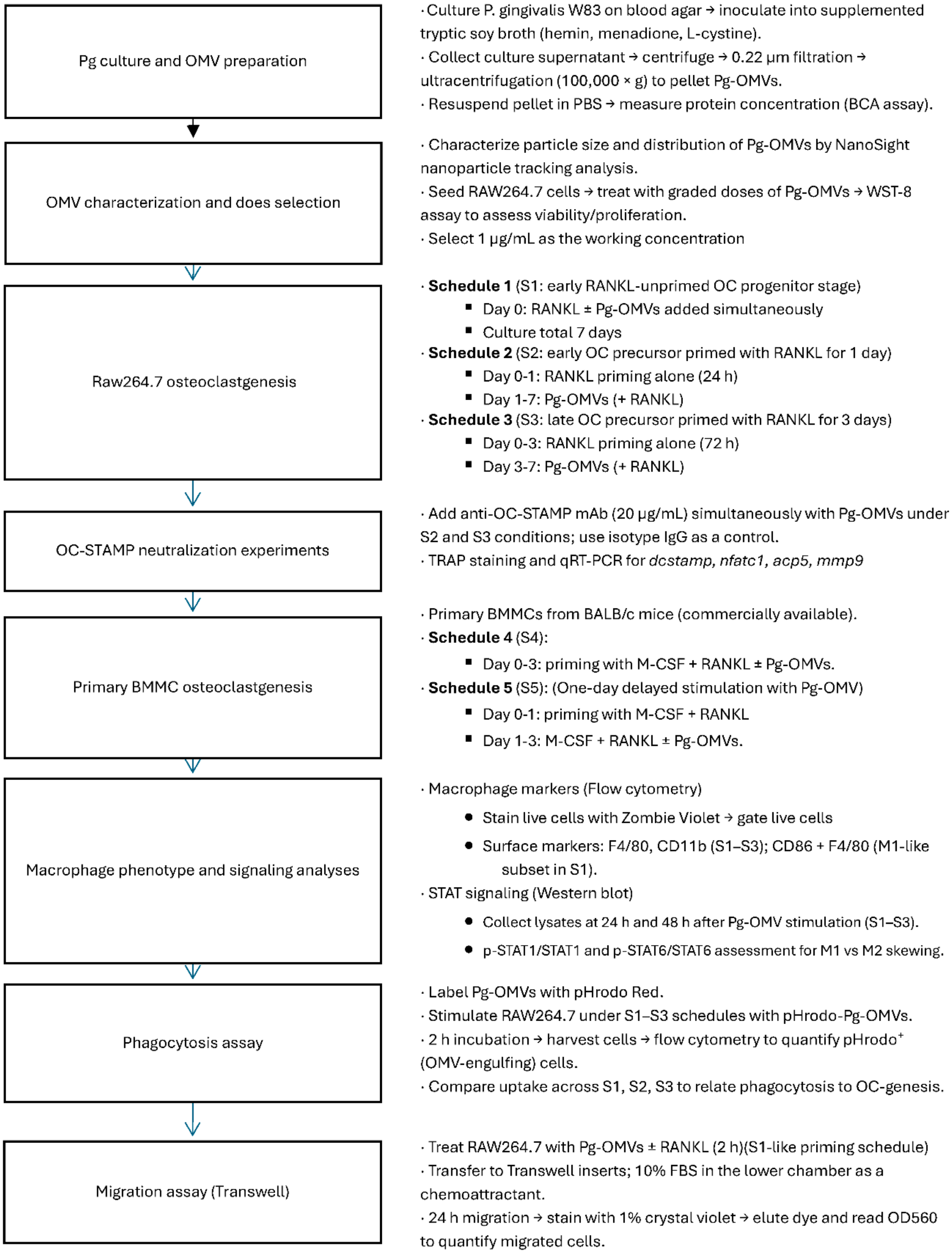

Figure S1. Experimental workflow illustrating the experiments performed this study. Flowchart summarizing the experimental design used to assess the stage-dependent effects of *Pg*-OMVs on osteoclast differentiation. OC-genesis was examined under three distinct treatment schedules, each modeling a different differentiation stage, and followed by additional analyses to determine how *Pg*-OMVs regulate the osteoclastogenic process.

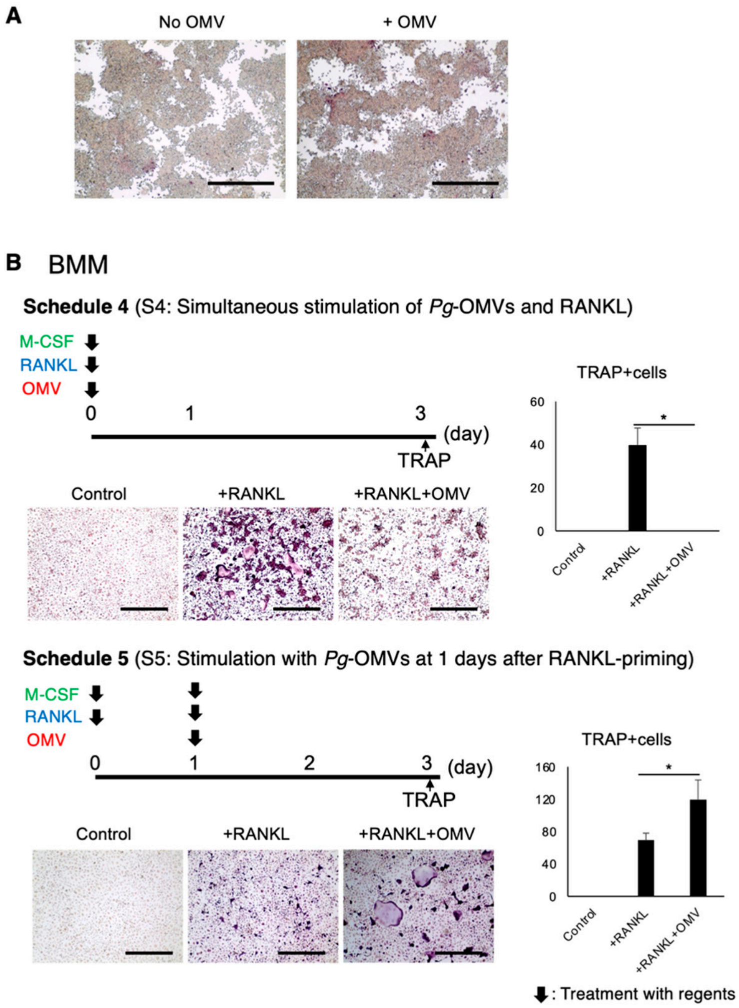

Figure S2. Effects of *Pg*-OMVs on OC-genesis from RAW264.7 cells and bone marrow–derived monocytes (BMMs). (A) TRAP staining of RAW264.7 cells stimulated with RANKL in the presence or absence of *Pg*-OMVs. Representative images illustrate that *Pg*-OMVs alone did not generate multinucleated osteoclasts, but modulated RANKL-driven differentiation. (B) TRAP staining of bone marrow–derived monocytes (BMMs) subjected to two schedules: Schedule 4 (S4), simultaneous stimulation with M-CSF, RANKL, and *Pg*-OMVs; and Schedule 5 (S5), 24 h pre-priming with M-CSF and RANKL followed by *Pg*-OMVs stimulation. Quantification reveals that simultaneous exposure (S4) suppresses osteoclast formation, whereas delayed *Pg*-OMVs stimulation (S5) enhances multinucleated osteoclast generation. These results confirm that the stage-dependent effects of *Pg*-OMVs observed in RAW264.7 cells are reproducible in primary BMM cultures. Images from the samples were obtained via EVOS™ XL Core Imaging System. Results were presented as the means ± SD (n=3). Experiments were independently repeated three times with similar results. Statistical analyses were performed using Kruskal-Wallis followed by Steel-Dwass's post-hoc test for multiple comparisons. *: $p<0.05$.

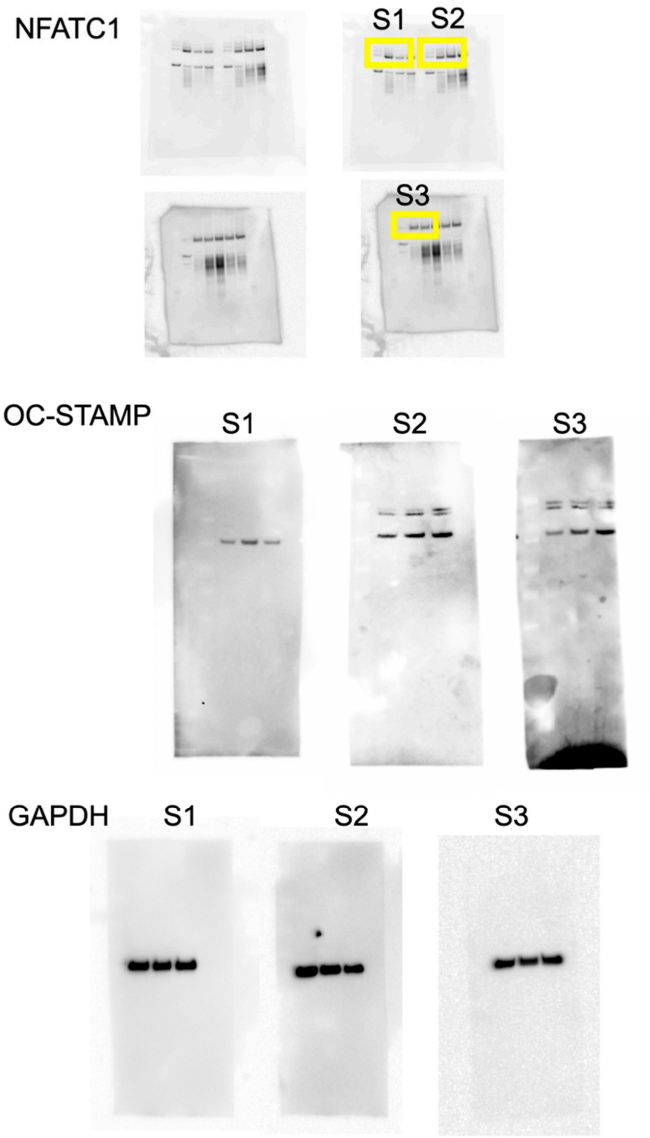

Figure S3. Original membrane images of Western blotting. Representative original Western blot images for NFATC1, OC-STAMP, and GAPDH. The area enclosed by yellow boxes indicate the portions that were cropped for figure preparation. The images were obtained from using the Azure C400.

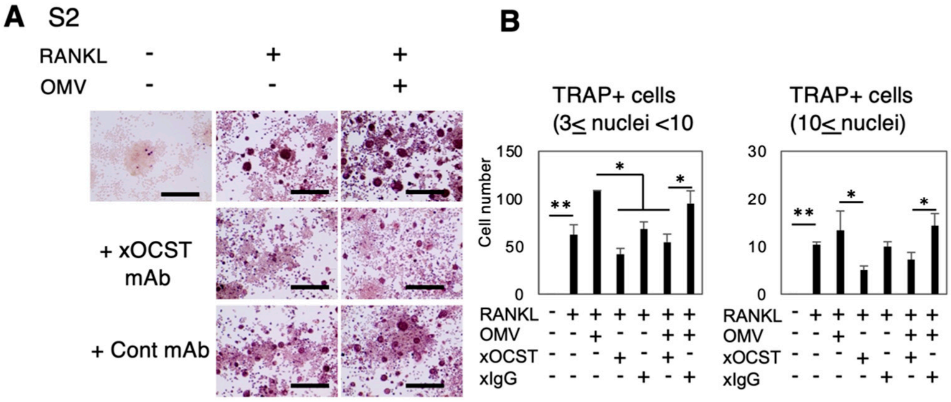

Figure S4. Neutralization of OC-STAMP suppresses *Pg*-OMVs–induced OC-genesis under Schedule 2. (A) Representative TRAP staining of RAW264.7 cells pre-primed with RANKL (S2) and then stimulated with *Pg*-OMVs, in the presence of either anti-OC-STAMP monoclonal antibody (xOC-STAMP mAb) or isotype control IgG. Images from the samples were obtained via EVOS™ XL Core Imaging System. (B) Quantification of TRAP-positive osteoclasts with ≥3 or ≥10 nuclei demonstrates that OC-STAMP blockade significantly reduced the number of large multinucleated osteoclasts induced by *Pg*-OMVs. These findings extend the results of Figure 3 (S3 condition) and support that OC-STAMP is a critical fusogen mediating *Pg*-OMVs–enhanced osteoclast fusion. Results were presented as the means ± SD (n=3). Experiments were independently repeated three times with similar results. Statistical analyses were performed using Kruskal-Wallis followed by Steel-Dwass's post-hoc test for multiple comparisons. *: $p<0.05$, **: $p<0.01$.

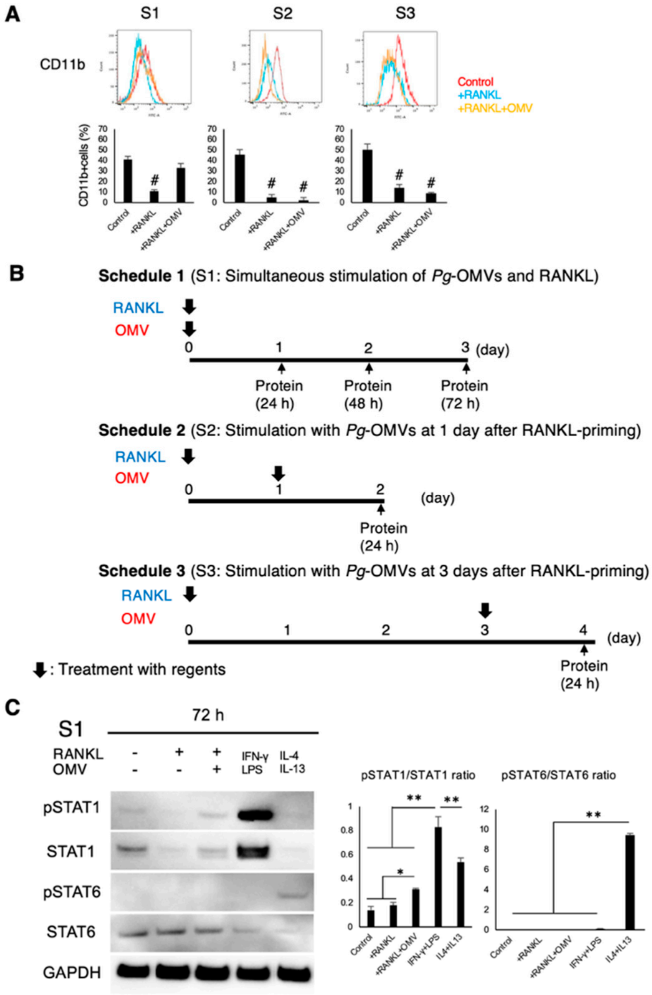

Figure S5. *Pg*-OMV effects on CD11b expression and STAT signaling at 72 h. (A) Flow cytometry analysis of CD11b expression on RAW264.7 cells stimulated with RANKL ± Pg-OMVs under S1, S2, or S3 conditions. Results show that simultaneous stimulation (S1) increased CD11b expression, whereas delayed stimulation (S2, S3) reduced expression compared to RANKL alone. The images were generated with FlowJo software (ver 11.11.1.0). (B) Western blot analysis of STAT1 and STAT6 phosphorylation after 72 h stimulation. Densitometry shows enhanced STAT1 phosphorylation in S1 compared to RANKL alone, consistent with induction of M1-like polarization. In contrast, *Pg*-OMVs reduced STAT1 activation in S2 and S3. STAT6 phosphorylation was undetectable under all conditions. (C) Quantification of pSTAT1/STAT1 and pSTAT6/STAT6 ratios confirms that *Pg*-OMVs modulate STAT1 signaling in a timing-dependent manner, biasing toward M1 polarization only under early (S1) co-stimulation. The images were obtained from using the Azure C400. Results were presented as the means ± SD (n=3). Experiments were independently repeated three times with similar results. Statistical analyses were performed using Kruskal-Wallis followed by Steel-Dwass's post-hoc test for multiple comparisons. *: p<0.05, **: p<0.01, #: p<0.05, vs control.

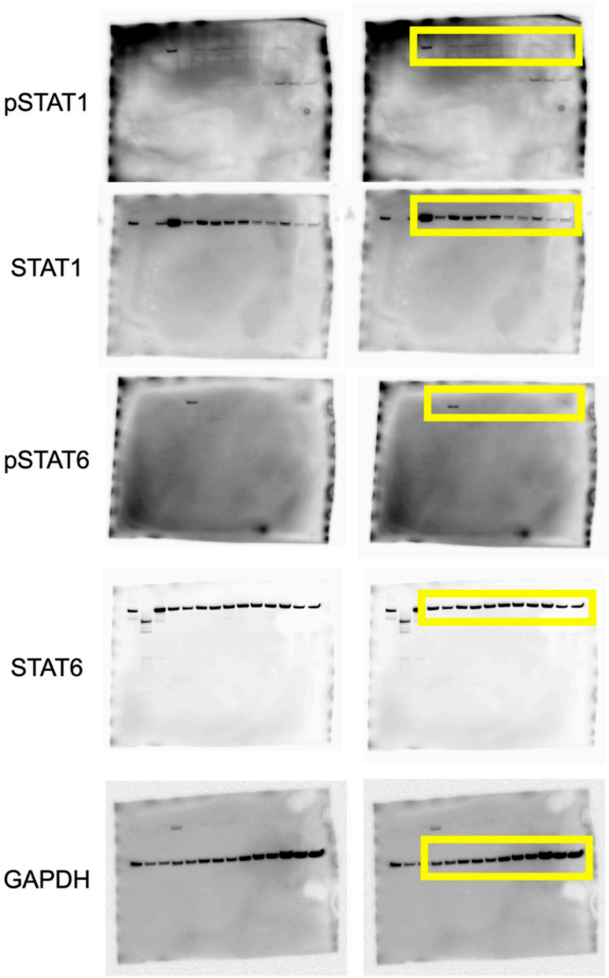

Figure S6. Original membrane images of Western blotting. Representative original Western blot images for pSTAT1, STAT1, pSTAT6, STAT6, and GAPDH. The yellow boxes indicate the regions that were cropped for the final figures. The images were obtained from using the Azure C400.

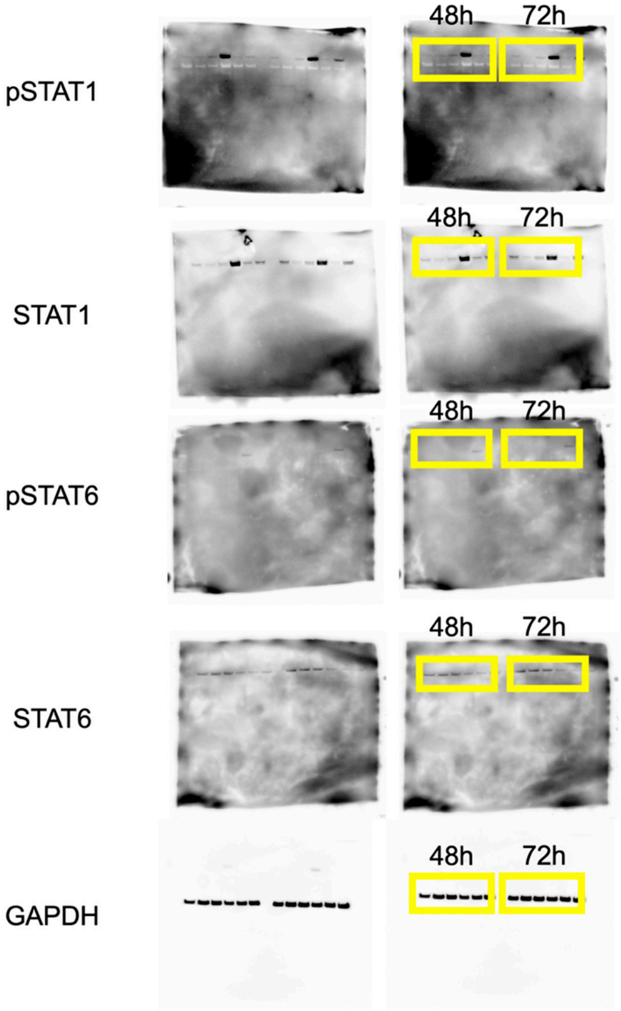

Figure S7. Original membrane images of Western blotting. Representative original Western blot images for pSTAT1, STAT1, pSTAT6, STAT6, and GAPDH. The yellow boxes indicate the regions that were cropped for the final figures. The images were obtained from using the Azure C400.

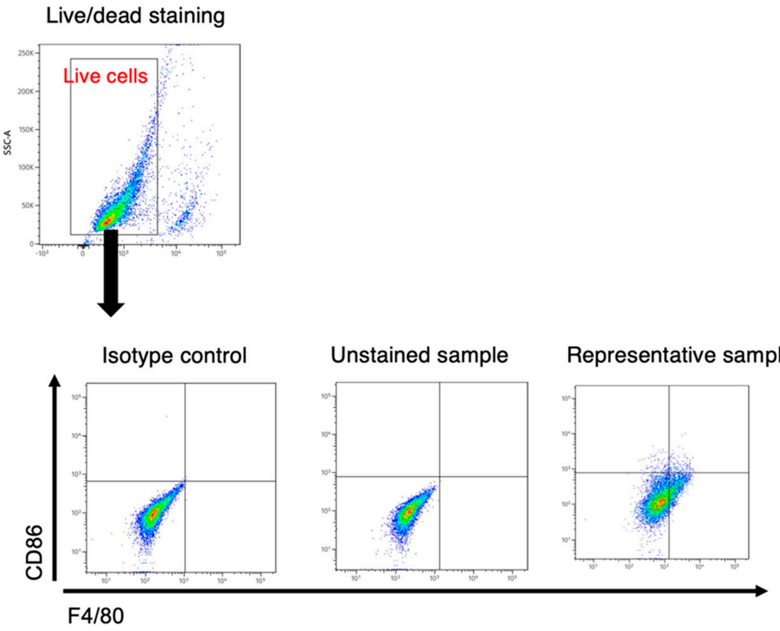

Figure S8. Gating strategy for identifying M1-like macrophages in flow cytometry analysis. Live cells were first selected by excluding Zombie Violet–positive cells, after which M1-like macrophages were defined as the CD86^{+}F4/80^{+} population. The images were generated with FlowJo software (ver 11.11.1.0).
